# Comparative transcriptomic analysis of silkworm^*Bmovo*-1^ and wild type silkworm ovary

**DOI:** 10.1038/srep17867

**Published:** 2015-12-08

**Authors:** Renyu Xue, Xiaolong Hu, Liyuan Zhu, Guangli Cao, Moli Huang, Gaoxu Xue, Zuowei Song, Jiayu Lu, Xueying Chen, Chengliang Gong

**Affiliations:** 1 School of Biology & Basic Medical Science, Soochow University, Suzhou 215123, China; 2 National Engineering Laboratory for Modern Silk, Soochow University, Suzhou, PR China; 3 Suzhou Zhenhua Middle School, Suzhou 215006, China

## Abstract

The detailed molecular mechanism of *Bmovo*-1 regulation of ovary size is unclear. To uncover the mechanism of *Bmovo*-1 regulation of ovarian development and oogenesis using RNA-Seq, we compared the transcriptomes of wild type (WT) and *Bmovo*-1-overexpressing silkworm (silkworm^+*Bmovo*-1^) ovaries. Using a pair-end Illumina Solexa sequencing strategy, 5,296,942 total reads were obtained from silkworm^+*Bmovo*-1^ ovaries and 6,306,078 from WT ovaries. The average read length was about 100 bp. Clean read ratios were 98.79% for silkworm^+*Bmovo*-1^ and 98.87% for WT silkworm ovaries. Comparative transcriptome analysis showed 123 upregulated and 111 downregulated genes in silkworm^+*Bmovo*-1^ ovaries. These differentially expressed genes were enriched in the extracellular and extracellular spaces and involved in metabolism, genetic information processing, environmental information processing, cellular processes and organismal systems. *Bmovo*-1 overexpression in silkworm ovaries might promote anabolism for ovarian development and oogenesis and oocyte proliferation and transport of nutrients to ovaries by altering nutrient partitioning, which would support ovary development. Excessive consumption of nutrients for ovary development alters nutrient partitioning and deters silk protein synthesis.

The domesticated silkworm *Bombyx mori* is an economically important sericultured insect. Important genes in the economic traits of silkworms include genes in silk protein synthesis, development and metamorphosis, sex determination and resistance to pathogens. Ovary development, oogenesis and silk synthesis are decisive factors for sericulture. To clarify the regulatory mechanism of silkworm ovarian development, we characterized splice variants of silkworm *Bmovo* genes in the ovary. Overexpression of the splice variant *Bmovo*-1 in silkworm gonads increases oviposition number and elevates the trehalose content of hemolymph and ovaries^1^. Based on previous results, we concluded that *Bmovo*-1 contributes to ovary development and oviposition number in silkworms and improves protein synthesis. *Bmovo*-1 overexpression in ovaries is hypothesized to improve ovary anabolism; elevate ovary proliferation and survival; and promote the transport of nutrients from hemolymph to ovaries, leading to larger ovarian sizes, improved egg productivity, and decreased silk protein synthesis^1^. These findings suggest a new strategy of pest control could be developed by genetically manipulating ovaries.

Insect genomes including *Lepidoptera*, *Coleoptera*, *Hymenoptera*, *Hemiptera*, *Diptera*, *Anoplura* and *Drosophila* contain *ovo*-like genes. Drosophila ovo is well characterized, but few ovo genes are reported in non-*Drosophila* except for *Bactrocera oleae*^2^. *Ovo* genes encode C_2_H_2_ zinc-finger transcription factors in flies, nematodes, mice and humans^3^,^4^,^5^,^6^,^7^,^8^ and are required for germ cell and epidermal development^6^,^9^,^10^. In *Drosophila*, *ovo-B* and *ovo-A* isoforms are produced by mRNA alternative splicing^11^,^12^,^13^. OVO-B is a transcriptional activator and OVO-A is a transcription suppressor^14^. OVO-B positively regulates the *ovarian tumor* (*otu*) promoter and OVO-A negatively regulates a target promoter^3^,^11^,^14^,^15^. *Otu* is strongly upregulated by OVO-B, and positively downregulated by OVO-A expression, which is located in a target locus directly downstream^16^. The *otu* locus is required for the regulation of oogenesis and germline sexual identity by *Sxl*^17^. OVO-B is indispensable for female germline development and epidermis. Elevated expressions of ovo-A results in maternal-effect lethality while the absence of ovo-A results in sterility. Thus, *ovo* is implicated in oogenesis and female germ-line sex determination^15^.

*B. mori* is a well-established model *Lepidopteran* insect with many fully characterized genes related to development, growth, metamorphosis, fibroin synthesis and innate immunity response. However, little is known about the detailed mechanism by which *Bmovo*-1 regulates ovarian development. In this paper, to understand the molecular mechanisms of *Bmovo*-1 regulation, transcriptional changes in the ovary were investigated by RNA-Seq after upregulating *Bmovo*-1 expression in ovaries. In ovaries of silkworms overexpressing *Bmovo*-1 (silkworm^+*Bmovo*-1^), 123 genes were upregulated genes and 111 were downregulated. *Bmovo*-1 overexpression in ovaries is suggested to elevate anabolism ovaries, increase oocyte proliferation, and facilitate transportation of nutrients to ovaries. These processes result in enlarged ovary sizes increased oocyte proliferation, and weakened silk protein synthesis.

## Results

### Transcription of genes related to gonad development in silkworm^+*Bmovo*-1^ ovaries by quantitative real-time PCR (qPCR)

**Figure 1:**
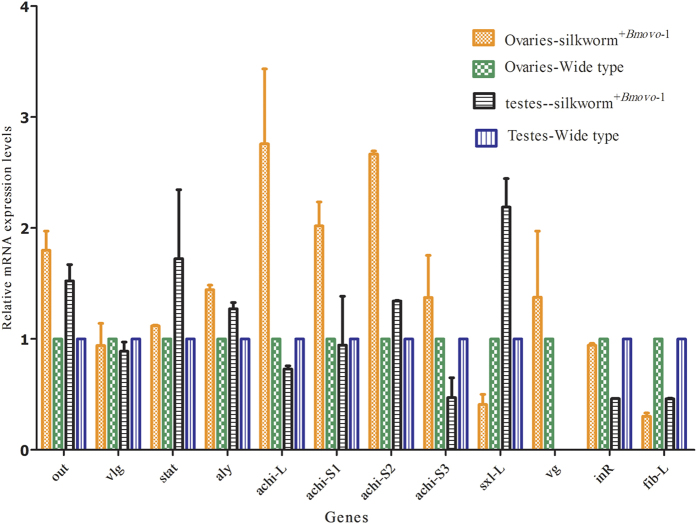
Effect of overexpressing *Bmovo*-1 on expression of genes in gonads. *Otu*: ovarian tumor, achi including *achi*-L, *achi*-S1, and *achi*-S2: *achintya*, *sxl*: sex-lethal-L, *vlg*: vasa-like, *stat*: signal transducer and activator of transcription, *aly*: always early, *vg*: vitellogenin, *inR*: insulin receptor and fib-L: fibroin L.

To determine if upregulation of *Bmovo*-1 expression specifically in gonads affected genes related to gonad development, several genes were selected for validation. In silkworm^+*Bmovo*-1^ ovaries, transcription increased for *otu*, *achintya* and *sxl*. Transcription of *vlg*, *stat*, *aly*, *vg* and *inR* was not changed compared to levels in WT silkworm ovaries (Fig. 1). In silkworm^+*Bmovo*-1^ testes, transcription *otu*, *stat*, and *sxl*-S increased and transcription of *achi*-S3 and *inR* decreased; expression of *vlg*, *aly*, *achi*-L, *achi*-S1 and *achi*-S2 were less affected (Fig. 1). Transcription of *fib*-L was reduced in female and male larval silk glands (Fig. 1).

### Identification of differentially expressed genes using RNA-seq

To understand the molecular mechanism by which *Bmovo*-1 regulates ovarian development in silkworms, comparative transcriptomic analysis of ovaries of transgenic silkworm^+*Bmovo*-1^ and WT was carried out by RNA-Seq. Using a pair-end Illumina Solexa sequencing strategy, 5,296,942 total reads from silkworm^+*Bmovo*-1^ ovaries and 6,306,078 total reads from WT ovaries were obtained. The average read length was about 100 bp. Clean reads ratios were 98.79% for silkworm^+*Bmovo*-1^ and 98.87% for WT. Ratios of sequenced genes with gene coverage between 90% and 100% were 38.61% for silkworm^+*Bmovo*-1^ and 43.78% for WT. Silkworm^+*Bmovo*-1^ ovaries had 123 upregulated and 111 downregulated genes false discovery rate (FDR <0.001) (Supplementary information Table 1). Of mapped genes, 29 (read numbers ≥3) were detected only in silkworm^+*Bmovo*-1^ ovaries (Supplementary information Table 2), while 61 (reads number ≥3) of the mapped genes were detected only in WT ovaries (Supplementary information Table 3).

### Functional annotation of Differentially expressed genes (DEGs)

**Figure 2:**
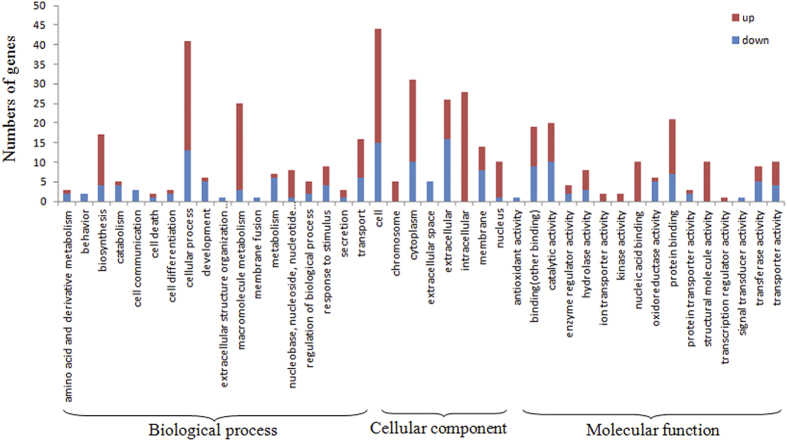
GO classification of upregulated and downregulated genes in silkworm^+*Bmovo*-1^ovaries. Genes were classified into cellular component, molecular function and biological process categories by WEGO according to GO terms.

**Figure 3:**
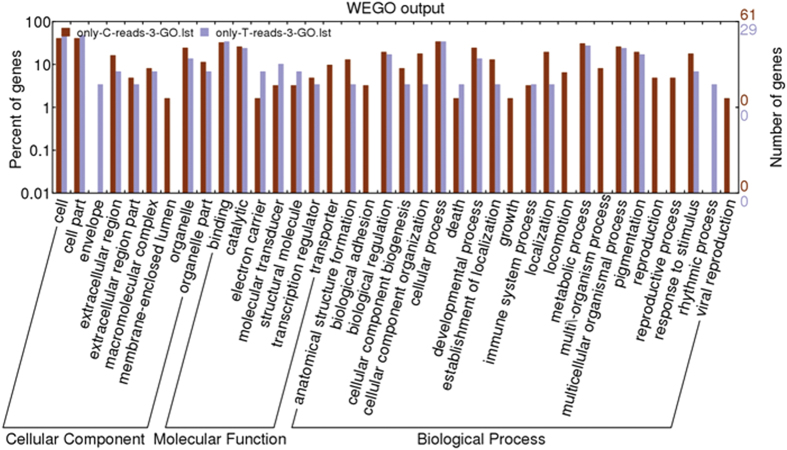
GO classification of genes expressed only in silkworm^+*Bmovo*-1^ or WT silkworm ovaries. Genes were classified into cellular component, molecular function and biological process categories by WEGO according to GO terms.

Upregulated and downregulated silkworm^+*Bmovo*-1^ ovary genes were annotated with Web Gene Ontology Annotation Plot (WEGO) (Fig. 2). Gene Ontology (GO) enrichment analysis showed that GO terms of DEGs relative to all genes were enriched mainly in extracellular and extracellular space (Supplementary information Table 4). In addition, 11 cellular component, 15 biological process and 6 molecular function genes were expressed only in silkworm^+*Bmovo*-1^ ovaries and 11 cellular components, 22 biological process and 7 molecular function genes were expressed only in WT ovaries (Fig. 3). To identify the biological pathways that are active in silkworm^+*Bmovo*-1^ ovaries, we mapped DEGs to reference canonical pathways in Kyoto Encyclopedia of Genes and Genomes (KEGG)^18^,^19^. KEGG orthology analysis of the DEGs is in Supplementary information Tables 5 and 6. In silkworm^+*Bmovo*-1^ ovaries, 16 upregulated genes were in metabolism, 21 in genetic information processing, 4 in environmental information processing, and 2 each were in cellular processes and organismal systems. In metabolism regulation, five upregulated genes were in both energy and nucleotide metabolism, one each in amino acid metabolism and metabolism of cofactors and vitamins and two each in carbohydrate metabolism, metabolism of other amino acids and xenobiotics biodegradation and metabolism. In genetic information processing pathways, four upregulated genes were in transcription, 12 in translation and folding, and 6 in sorting and degradation. In environmental information processing pathways, one upregulated gene was in membranes and two were in signal transduction. Two genes in transport and catabolism of cellular processes were upregulated. Among all upregulated genes, only one each was related to immune, endocrine and nervous systems.

**Figure 4:**
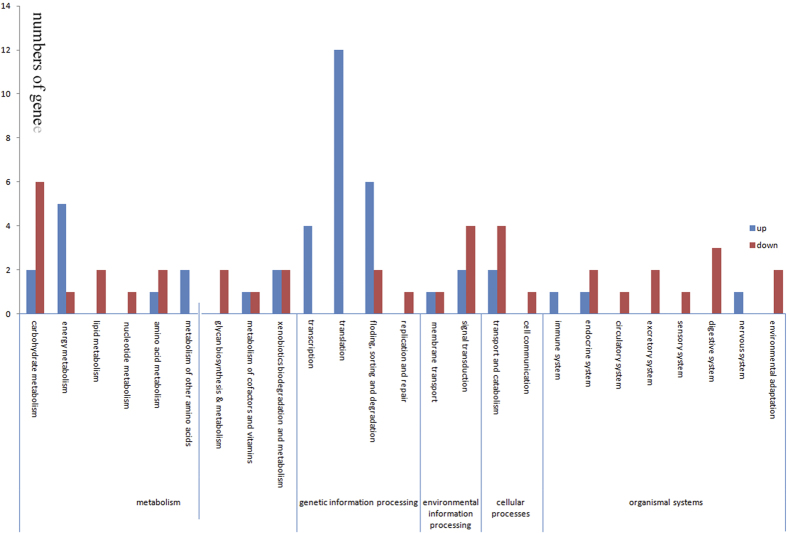
KEGG orthology classification of DEGs in silkworm^+*Bmovo*-1^ and WT silkworm ovaries.

Of the downregulated genes in silkworm^+*Bmovo*-1^ ovaries, ten were linked to metabolism, three to genetic information processing, three to environmental information processing, five to cellular processes and six to organismal systems. Among metabolism genes, six were in carbohydrate metabolism; one each in energy metabolism, nucleotide metabolism and metabolism of cofactors and vitamins; and two each in lipid metabolism, amino acid metabolism, glycan biosynthesis and metabolism, and biodegradation. In genetic information processing, two downregulated genes were related to folding, sorting and degradation, and only one was associated with replication and repair. In environmental information processing, an ATP-binding cassette, subfamily C (CFTR/MRP) and member 4 were related to ABC transporters of membrane transport. Four genes were linked to signal transduction in the Wnt, notch, calcium and phosphatidylinositol signaling pathways. In cellular processes, four genes were related to endocytosis, lysosomes and peroxisomes in transport and catabolism and a phosphatidylinositol phospholipase C beta was involved in gap-junction cell communication. In downregulated genes in organismal systems, two each were related to the endocrine system, excretory system, sensory system and environmental adaptation; one was related to the circulatory system; and three to the digestive system (Fig. 4).

Among expressed genes detected only in silkworm^+*Bmovo*-1^ ovaries, four genes were involved in metabolism (Supplementary information Table 7), with two each involved in carbohydrate metabolism, lipid metabolism, metabolism of cofactors and vitamins and xenobiotics biodegradation and metabolism; and one each in energy and nucleotide metabolism.

### qPCR verification of DEGs

**Figure 5:**
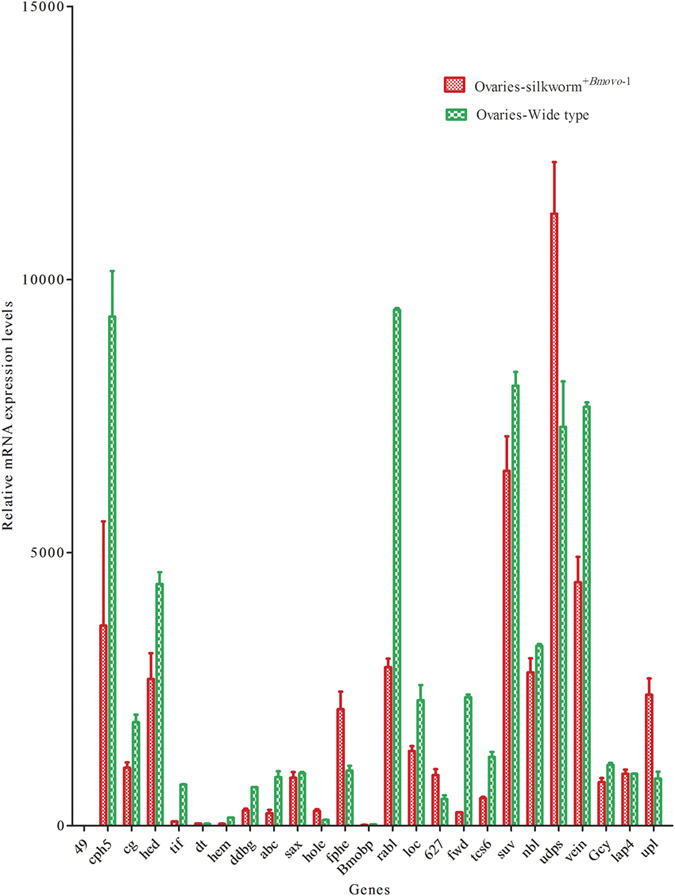
Validation of RNA-seq results by qPCR.

To validate the RNA-Seq data, expression relative to the housekeeping actin *A3* genes was determined by qPCR for 25 randomly selected genes. Transcription of the selected genes as determined by qPCR was similar to the RNA-Seq results (Fig. 5). This result suggested that the RNA-Seq data were credible.

## Discussion

Our previous study found that overexpression of *Bmovo*-1 in silkworm ovaries regulates gonadal size^1^. To understand this regulatory pathway, we determined the trehalose content in silkworm hemolymph and ovaries. Trehalose levels increased by 11.37% in the hemolymph and 10.56% in the ovaries of silkworm^+*Bmovo*-1^ compared with WT silkworms. No significant difference in trehalase activity was found in the hemolymph of silkworm^+*Bmovo*-1^ compared to WT. Therefore, upregulation of *Bmovo*-1 in the gonads of transgenic silkworms elevated the trehalose contents of hemolymph and ovaries. We concluded that *Bmovo*-1 was associated with protein synthesis and contributed to the development of silkworm ovaries^1^. However, the molecular mechanisms by which *Bmovo*-1 regulates gonad development are still obscure. Using silkworms overexpressing *Bmovo*-1 in the gonads, we used qPCR to estimate the relative expression of *otu*, *vlg*, *stat*, *aly*, a*chi-L*, *achi-S1*, *achi-S2*, *achi-S3*, *sxl-L*, *vg* and *inR*, which are associated with reproductive development in gonads, and the fib-L gene in silk glands. Transcription of *otu* and *sxl* increased in ovaries, suggesting that Bmovo-1 acted similarly to ovo-B in *Drosophila* and might be a transcriptional activator that regulated *otu* and *sxl* transcription in the female silkworm germline. In addition, overexpression of *Bmovo-1*, especially in silkworm gonads, decreased *fib*-L transcription in silk glands, reducing cocoon shell weight^1^. Germline-specific expression of *ovo* in *Drosophila* females correlates with function in oogenesis. This expression, however, is also observed in males that do not require *ovo*^[11,14]^. In silkworm^+*Bmovo*-1^, testis weight decreased slightly^1^; nevertheless, transcription genes associated with spermatogenesis (*aly*, *achi*) was not significantly changed, suggesting that *Bmovo-1* had no effect on spermatogenesis.

To globally analyze the effect of overexpressing *Bmovo*-1 on gonadal development, comparative transcriptomic analysis was performed using silkworm^+*Bmovo*-1^ and WT ovaries with RNA-Seq. Silkworm^+*Bmovo*-1^ ovaries had 123 upregulated genes and 111 downregulated genes. GO terms of the DEGs relative to all genes were enriched in extracellular and extracellular space. These results provide important clues for understanding the integrated effect of upregulated *Bmovo*-1 on silkworm gonad development. We propose that many genes and metabolism pathways related to nutrient metabolism and proteins synthesis were affected by *Bmovo*-1 upregulation in the ovary. Our analysis identified many genes including for 30 K proteins, apolipophorin III and adducin that were regulated by overexpressing *Bmovo*-1 in the ovary. Many nucleotide metabolism pathways, transcription and translation pathways and Wnt signaling were regulated by overexpressing *Bmovo*-1 in ovaries.

The 30 K proteins are involved in the innate immune response and transport of lipid and sugar^20^. *B. mori* contains a group of homologous proteins of approximately 30 kDa termed *B. mori* low molecular weight lipoproteins (Bmlps). The increased transcription of 30 K proteins 11, 3, 7 and low molecular weight 30 kDa lipoprotein PBMHP-6 precursor genes in the ovary might have promoted transport of lipid and sugar from the hemolymph to the ovary. When *Bmovo*-1 is overexpressed in silkworms, trehalose content is elevated by 11.37% in hemolymph and 10.56% in ovaries^1^. In silkworms, the ovary absorbs nutrients from the hemolymph for egg development. Another gene related to lipid transport is apolipophorin III^21^, which was also upregulated in silkworm^+*Bmovo*-1^ ovaries. Promoting the transport of lipid and sugar is conducive to ovarian development. Excessive consumption of nutrients for ovary development alters nutrient partitioning and deters silk protein synthesis.

*Drosophila* adducin (Ovhts) is predicted to have actin-binding function and is involved in female germline ring canal formation and ovarian fusome organization. Reducing Ovhts delays the transition from fusome-containing cells to cells with ring canals^22^. Expression of the silkworm *adducin* gene was downregulated in silkworm^+*Bmovo*-1^ ovaries. This finding might indicate delays in fusome organization during oocyte differentiation in *B. mori*. Ceramide, sphingosine and sphingosine-1-phosphate are directly regulated by ceramidase. These bioactive lipids mediate cell proliferation, differentiation, apoptosis, adhesion, and migration^23^. In silkworm^+*Bmovo*-1^, expression of the *ceramidase* gene decreased in ovaries, influencing oogenesis.

Juvenile hormone (JH) and 20-hydroxyecdysone (20E) are gonadotropic in adult insects^24^. Increasing JH titers in *Drosophila virilis* before the beginning of starvation sharply increases fertility measured as number of laid eggs and number of progeny within the first 24 h after starvation termination^25^. All JH actions begin as transport of JH in the hemolymph as a complex with JH-binding protein (JHBP) to target tissues^26^,^27^. Complex formation protects the chemically labile JH against nonspecific enzymatic degradation and sequestration. Increased JHBP expression is proposed to be caused by overexpression of *Bmovo*-1, elevating silkworm egg productivity. Moreover, expression of sex-specific storage-protein genes was elevated in silkworm^+*Bmovo*-1^ gonads, leading to increased egg productivity. In general, we conclude that transport of nutrients to ovaries is promoted by overexpression of *Bmovo*-1 in gonads, increasing egg productivity and leading to reduced silk protein synthesis.

KEGG orthology analysis showed that both upregulated and downregulated genes in silkworm^+*Bmovo*-1^ ovaries were related to pathways whose interaction might influence oogenesis and egg development. A ribose-phosphate pyrophosphokinase gene (prsA), which was preeminent in silkworm^+*Bmovo*-1^. PrsA is involved in the pentose phosphate pathway, a substitute to glycolysis, and purine metabolism. While the pentose phosphate pathway involves glucose oxidation, its primary role is anabolic rather than catabolic^28^. Also elevated were DNA-directed RNA polymerase I subunit RPA43 and DNA-directed RNA polymerases I, II, and III subunit RPABC1 involved in nucleotide metabolism pathways and enhanced purine metabolism or pyrimidine metabolism. In the purine metabolism pathway, the retinal rod rhodopsin-sensitive cGMP 3′, 5′-cyclic phosphodiesterase converts 3′, 5′-cyclic GMP to GMP. All these increased genes are involved in the synthesis of nucleotides and nucleic acids in the ovary for oogenesis and egg development.

Glycine cleavage system H protein is involved in glyoxylate and decarboxylate metabolism, cleaves glycine to NH3 and provides hydroxypyruvate and glyoxylate, so biosynthesis of carbohydrates from fatty acids or two-carbon precursors is promoted in ovaries when glycine cleavage system H protein is enhanced. NADH dehydrogenase (ubiquinone) Fe-S protein 5, NADH dehydrogenase (ubiquinone) 1 alpha subcomplex 7, NADH dehydrogenase (ubiquinone) 1 beta subcomplex 2, F-type H^+^-transporting ATPase subunit 6 and F-type H^+^-transporting ATPase subunits are involved in oxidative phosphorylation, which releases energy by oxidation of nutrients to form ATP. These increased proteins could supply extra energy for oogenesis and egg development.

Modulating rRNA synthesis is proposed to foster changes in cell fate, growth, and proliferation of female *Drosophila* germline stem cells (GSCs) and their daughters^29^. We found that genes relevant to transcription pathways that were upregulated in silkworm^+*Bmovo*-1^ were DNA-directed RNA polymerases I, II, and III subunit RPABC1, DNA-directed RNA polymerase I subunit RPA43, U6 snRNA-associated Sm-like protein LSm8 and coiled-coil domain-containing protein 12. Upregulation of these genes increased transcription of genes, simultaneously with elevated expression of 11 ribosomal proteins and ribonucleoprotein complex subunit 3, which is involved in ribosome biogenesis. Genes of large and small subunit ribosomal proteins were also elevated. The elevation of transcription and translation would benefit increased egg productivity.

The expression genes for protein transport protein SEC61 subunit gamma and related proteins, signal recognition particle subunit SRP9, signal peptidase complex subunit 1 and crystalline alpha B, which are involved in protein processing in the endoplasmic reticulum were increased. This would increase the ability of the ovary to provide proteins for developing oocytes. Mannosyl-oligosaccharide alpha-1,2-mannosidase, which catalyzes the removal of three distinct mannose residues from peptide-bound Man(9)-GlcNAc(2) oligosaccharides, is involved in N-glycan biosynthesis and N-glycan biosynthesis and protein processing in the endoplasmic reticulum. Endoplasmic reticulum lectin 1 is a receptor that recognizes hydrolytic enzymes containing mannose-6-phosphate and targets these proteins for delivery to lysosomes. The expression of these two genes was downregulated in silkworm^+*Bmovo*-1^ ovaries, which would reduce the degradation of proteins and improve egg yield.

ABC transporters are transmembrane proteins that use ATP hydrolysis to perform biological processes including translocation of substrates across membranes^30^,^31^. In silkworm^+*Bmovo*-1^ ovaries, expression of the ATP-binding cassette subfamily D (ALD) member 3 gene was upregulated and the ATP-binding cassette subfamily C (CFTR/MRP) member 4 gene was downregulated. ALD member 3 is involved in the peroxisome, whose major function is breakdown of very long-chain fatty acids through beta-oxidation^32^. CFTR/MRP member 4 is involved in ion transport, cell-surface receptors and toxin secretion^31^, suggesting that membrane transport is influenced by regulating expression of these genes in silkworm^+*Bmovo*-1^ ovaries, affecting ovary development and oogenesis.

Mitogen-activated protein kinases (MAPKs) are involved in programs such as cell proliferation, differentiation, movement, and death^33^,^34^. Cyclic AMP-dependent transcription factor ATF-2 binds to cAMP-responsive elements. The protein is a homodimer or heterodimer with c-Jun and stimulates CRE-dependent transcription^35^. ATF2 is involved in the MAPK signaling pathway and the PI3K-Akt signaling pathway, an intracellular signaling pathway important in apoptosis. In many cancers, this pathway is overactive, reducing apoptosis and allowing proliferation^36^. The *Drosophila* p38 MAPK pathway is essential for oogenesis^37^. *Drosophila* ATF-2 is directly phosphorylated by p38b. Genetic analysis indicates that *Drosophila* ATF-2 acts in the *Drosophila* p38 signaling pathway and is critical for the p38-mediated stress response^38^. Akt determines cell fate through inhibition of the PERK-eIF2α phosphorylation pathway^39^. We found that the MAPK and PI3K-Akt signaling pathways were elevated in silkworm^+*Bmovo*-1^ ovaries, suggesting that these pathways promoted the proliferation and survival of oocytes, increasing egg yield.

KEGG analysis shows that phosphatidylinositol phospholipase C beta is involved in the Wnt signaling pathway (noncanonical Wnt/calcium pathway), which regulates calcium in cells, calcium signaling pathways, phosphatidylinositol signaling systems and gap junctions. The extracellular matrix pattern of *Drosophila* ovary follicle cells is regulated by several signaling pathways, including the Wnt, calcium and phosphatidylinositol signaling pathways^40^. We found that expression of the phosphatidylinositol phospholipase C beta gene in silkworm^+*Bmovo*-1^ ovaries was downregulated, suggesting that ovary development, oogenesis and egg yield in silkworms was affected by expression of the phosphatidylinositol phospholipase C beta gene. The notch signaling pathway is a highly conserved celluar signaling system present in most multicellular organisms. In *Drosophila melanogaster*, JAGGED/Serrate is a Notch ligand. The Notch signaling pathway has multiple functions in *Drosophila* oogenesis^41^. Ectopic or expanded activation of Notch signaling leads to formation of more cap cells and larger niches, which induces ectopic or more GSCs; conversely, decreased Notch signaling during niche formation results in reduced cap cell number and niche size and fewer GSCs^42^. We found that the jagged gene was downregulated in silkworm^+*Bmovo*-1^ ovaries, which might expand activation of the Notch signal, leading to increased egg yield. We found that expression of some genes was switched on or off in silkworm^+*Bmovo*-1^ ovaries. However, the exact interaction among these gene products in ovary development and oogenesis needs further study.

Generally, BmOVOs with zinc fingers but different N-termini are transcription factors. The expression patterns of *Bmovo* isoforms are distinctive among the four isoforms^1^. Ovary development and oogenesis are positively regulated by BmOVO-1, which decreases silk protein synthesis^1^. These might promote the proliferation and survival of oocytes, enhancing anabolism for ovary development and oogenesis, and facilitating nutrient transport from hemolymph to ovaries triggered by overexpression of *Bmovo*-1 in ovaries. These findings provide novel insights into the function of *Bmovo* and information for improving silk protein synthesis in silkworms. The findings also contribute to our understanding of the transport of nutrients between different tissues.

## Methods

### Animals

Transgenic silkworms^**+***Bmovo*-1^ overexpressing the *Bmovo*-1 gene in gonads were obtained as described^1^. Both transgenic and wild type (WT) silkworms (strain P50) were fed mulberry leaves (*Morus* sp.) and kept at 25 ± 1 °C at 70%–85% relative humidity and a 12 h light /12 h dark photoperiod.

### Dissection of the ovaries and testes and RNA extraction

The ovaries and testes were dissected from 3^rd^ day, 6^th^ day of fifth instar larvae and 2^nd^ day of pupae. Total RNAs were isolated from 20 ovaries of silkworm^**+***Bmovo*-1^ and WT silkworms, respectively, at the 3^rd^ day, 6^th^ day of fifth instar larvae and 2^nd^ day of pupae using RNAout Kit (Tiandz, Mianyan, China), followed by treatment with RNase-free DNaseI to remove possible contamination with genomic DNA according to the protocol. RNA quality was verified using agarose gel electrophoresis and NanoDrop ND-1000 Spectrophotometer (NanoDrop, Wilmington, DE, USA).

### Detection of genes related to gonad development by qPCR

Total RNA (1 μg) from ovaries or testes of the 3^rd^ day of fifth instar silkworms was used to synthesize first-strand cDNA using a SuperScript® III First-Strand Synthesis System (Invitrogen, Carlsbad, CA, USA). Prepared cDNA was used as a template for qPCR. *BmActin3* was used as the internal reference gene. Relative expression of genes ovarian tumor (*otu*), *achintya* (achi including *achi*-L, *achi*-S1, and *achi*-S2) sex-lethal-L (*sxl*), vasa-like (*vlg*), signal transducer and activator of transcription (*stat*), always early (*aly*), vitellogenin (*vg*), insulin receptor (*inR*) and fibroin L (fib-L). Our previous study found that *Bmovo*-1 overexpression in silkworm ovaries decreases silk protein synthesis^1^. Therefore, the *fib*-L gene was selected for analysis using the 2^–ΔΔCt^ method^43^. Experiments were performed with triplicate biological duplicates. Primers are in Supplementary information Table 8.

### Analysis of expression profile with RNA-Seq

MRNAs were purified using Micropoly (A) Purist^TM^ mRNA purification kits (Ambion, Woodward Austin, TX, USA) and cDNAs were synthesized by PrimeScript™ Reverse Transcriptase (TaKaRa, Dalian, China) following the methods of Ng^44^. Equal amounts of cDNA from different time periods were mixed and fragmented into 300–500 bp; cDNA libraries were constructed using TruSeq^TM^ DNA Sample Prep Kit-Set A (Illumina, San Diego, CA, USA) and amplified using TruSeq PE Cluster Kits (Illumina) after purification with Ampure beads (Agencourt, Beverly, MA, USA). Sequencing was with an Illumina Hiseq 2000 (Illumina).

### Bioinformatic analysis

Cellular components, molecular functions and biological processes were determined by GO database annotations (http://www.geneontology.org/). GO classification of matched proteins was with WEGO (http://wego.genomics.org.cn/). The signaling pathways of proteins were elucidated using the KEGG database (http://www.genome.jp/kegg/pathway.html).

### Analysis of expression abundance

Generated clean reads were mapped directly into deposited reference *B. mori* gene sequences in GenBank after eliminating low-quality sequences using TopHat software^45^. All read numbers of genes were converted into reads per kilobases per million reads (RPM) as described by Mortazavi *et al*.^46^. Expression abundant differences of genes were calculated using the MA-plot-based method with random sampling model of the DEGseq software package. Genes differentially expressed between two samples were identified at an FDR of 0.1%^47^. DEG enrichment relative to all genes in GO and KEGG pathways were analyzed using the hypergeometric distribution for each KEGG pathway and GO term^18^.

### Validation of RNA-seq data with qPCR

To validate the RNA-Seq data, the expression of 25 randomly selected *B. mori* relative to the housekeeping gene actin *A3* was determined by qPCR. Genes were uncharacterized LOC101735700 (49, XM_004929105.2), *B. mori* cuticular protein hypothetical 5 (cph5, NM_001173301.1), *B. mori* protein lap4 (cg, XM_012694181.1), interference hedgehog-like (hed, BGIBMGA008552), *B. mori* uncharacterized LOC101736962 (tif, XM_004923597.1), *B. mori* uncharacterized LOC101742051 (dt, XM_012689442.1), hemicentin-1-like (hem, BGIBMGA004887), *B. mori* putative uncharacterized protein DDB_G0271606-like (ddbg, gi 512937966), ABC transporter G family member 20-like (abc, XM_004933031.1), roundabout (sax, BGIBMGA000888-TA), *B. mori* nuclear pore complex protein Nup214-like (hole, XM_012692005.1), *B. mori* mRNA (fphe, AK385794.1), hypothetical protein (Bmobp, BGIBMGA010275-TA), ras-related protein Rab-24-like (rabl, XM_004922217.1), *B. mori* uncharacterized LOC101744394 (loc, gi 512892762), *B. mori* mRNA (627, BGIBMGA010627), hormone receptor 3 (fwd, NM_001043547.1), CCR4-NOT transcription complex subunit 6-like (tcs6, XM_004923523.1), ATP-dependent RNA helicase SUV3 homolog (suv, XM_012691746), *B. mori* neurobeachin-like (nbl, XM_012688376.1), *B. mori* UDP-glycosyltransferase UGT46A2 (udps, XM_012693087.1), protein vein-like (vein, BGIBMGA012742-TA), receptor type guanylyl cyclase (Gcy, BGIBMGA010092-TA), protein lap4-like (lap4, XM_004930615.1), and *B. mori* E3 ubiquitin-protein ligase RNF25-like (up, gi 512911275). Total RNA from silkworm^+*Bmovo*-1^ and WT ovaries was extracted using TRIzol (Invitrogen). Equal amounts of cDNAs from the 3^rd^ day, 6^th^ day of fifth instar larvae and 2^nd^ day of pupae were mixed for qPCR performed according to the manual for a CFX96 Touch™ Real-Time PCR Detection System (Bio-Rad, Hercules, CA, USA). Primers are in Supplementary information Table 8.

### Statistics

All data are presented as mean ± standard deviation (SD). Statistical differences were evaluated using Student’s *t*-test for unpaired samples. The level of statistically significant difference was set at *P < 0.05, **P < 0.01 and ***P < 0.001.

## Additional Information

**How to cite this article**: Xue, R. *et al*. Comparative transcriptomic analysis of silkworm*^Bmovo^*^-1^ and wild type silkworm ovary. *Sci. Rep*. **5**, 17867; doi: 10.1038/srep17867 (2015).

## Supplementary Material

Supplementary Table S1

Supplementary Table S2

Supplementary Table S3

Supplementary Table S4

Supplementary Table S5

Supplementary Table S6

Supplementary Table S7

Supplementary Table S8

Supplementary Information
