# Supplementary material for: Comparative transcriptomic analysis of silkwormBmovo-1 and wild type silkworm ovary
Source: Sci Rep. 2015 Dec 8;5:17867. doi: 10.1038/srep17867 (PMC4672304; doi:10.1038/srep17867)
Supplement: Supplementary Information [file srep17867-s9.doc]

**Comparative transcriptomic analysis of silkworm*^Bmovo^*^-1^ and wild type silkworm ovary**

Renyu Xue^a,b,1^, Xiaolong Hu^a,b,1^, Yuanli Zhu^a^, Guangli Cao^a,b^, Moli Huang^a^, Gaoxu Xue^a^, Zuowei Song^a^, Jiayu Lu^c^, Xueying Chen^c^, Chengliang Gong^a,b,*^

^a^School of Biology & Basic Medical Science, Soochow University, Suzhou 215123, China

^b^National Engineering Laboratory for Modern Silk, Soochow University, Suzhou, PR China

^c^Suzhou Zhenhua Middle School, Suzhou 215006, China

^1^These authors contributed equally to this work.

*Corresponding author: Tel.: +86-512-65880183; fax: +86-512-65880183.

E-mail address: gongcl@suda.edu.cn

**Supplemental Table legends**

Supplemental Table 1 The DEGs in the ovary between silkworm^+^*^Bmovo^*^-1^ and WT silkworm

Supplemental Table 2 The genes expression only detected in the ovary of silkworm^+^*^Bmovo^*^-1^

Supplemental Table 3 The genes expression only detected in the ovary of WT silkworm

Supplemental Table 4 The enriched GO terms of the DEGs relative to the entire genes

Supplemental Table 5 KEGG Orthology analysis of the upregulated expression genes in the ovary of silkworm^+^*^Bmovo^*^-1^

Supplemental Table 6 KEGG Orthology analysis of the downregulated expression genes in the ovary of silkworm^+^*^Bmovo^*^-1^

Supplemental Table 7 KEGG Orthology analysis of the expression genes only detected in the ovary of silkworm^+^*^Bmovo^*^-1^

Supplemental Table 8 The primers used in this paper
